# Essential Oil Molecules Can Break the Loop of Oxidative Stress in Neurodegenerative Diseases

**DOI:** 10.3390/biology12121504

**Published:** 2023-12-07

**Authors:** Enzo Spisni, Maria Chiara Valerii, Maria Lina Massimino

**Affiliations:** 1 Department of Biological, Geological, and Environmental Sciences, University of Bologna, 40126 Bologna, Italy; mariachiara.valerii2@unibo.it; 2 CIRI Life Sciences and Health Technologies, University of Bologna, 40126 Bologna, Italy; 3 Neuroscience Institute, Italian National Research Council (CNR), 35131 Padova, Italy

**Keywords:** neurodegenerative diseases, oxidative stress, essential oils, Alzheimer’s disease, Parkinson’s disease, amyotrophic lateral sclerosis

## Abstract

**Simple Summary:**

Neurodegenerative diseases (NDs) affect millions of people worldwide. The combination of genes and environment, associated with age, contributes to the risk of developing one of these diseases. NDs occur when neurons in the brain and in the peripheral nervous system lose function over time and ultimately die in some area of the brain. The mechanism of neuronal deaths is specific for each ND and may involve different nervous cell types, but always includes excessive oxidative stress and increased inflammatory responses. Although certain drugs may help relieve some of the physical or mental ND symptoms, sometimes slowing down their progression, no cure exists. This mini review focuses on the possibility to explore the efficacy of selected promising essential oil molecules to effectively counteract the oxidation and inflammation that occurs in NDs.

**Abstract:**

Essential oils (EOs) are mixtures of volatile compounds, extracted from aromatic plants, with multiple activities including antioxidant and anti-inflammatory ones. EOs are complex mixtures easy to find on the market and with low costs. In this mini narrative review, we have collected the results of in vitro and in vivo studies, which tested these EOs on validated models of neurodegeneration and in particular of the two main neurodegenerative diseases (NDs) that afflict humans: Alzheimer’s and Parkinson’s. Since EO compositions can vary greatly, depending on the environmental conditions, plant cultivar, and extraction methods, we focused our attention to studies involving single EO molecules, and in particular those that have demonstrated the ability to cross the blood–brain barrier. These single EO molecules, alone or in defined mixtures, could be interesting new therapies to prevent or slow down oxidative and inflammatory processes which are common mechanisms that contribute to neuronal death in all NDs.

## 1. Neurodegeneration and Oxidative Stress

Neurodegenerative diseases (NDs) are a major health problem worldwide that can be caused by genetic factors and ageing. Their increased prevalence in recent years can be related to an increased exposure of the population to extrinsic factors, collectively called exposome, that include diet, lifestyle, and environmental pollutants [1,2]. NDs are incurable and devastating neurological disorders defined by the progressive loss of neurons in different areas of the central nervous system (CNS), leading to cognitive, behavioral, motor, and sensory dysfunctions. Protein aggregation, chronic inflammation, and progressive neuronal loss are common signatures of many NDs, including Alzheimer’s disease (AD), Parkinson’s disease (PD), Huntington’s disease (HD), amyotrophic lateral sclerosis (ALS), and multiple sclerosis (MS).

**Figure 1:**
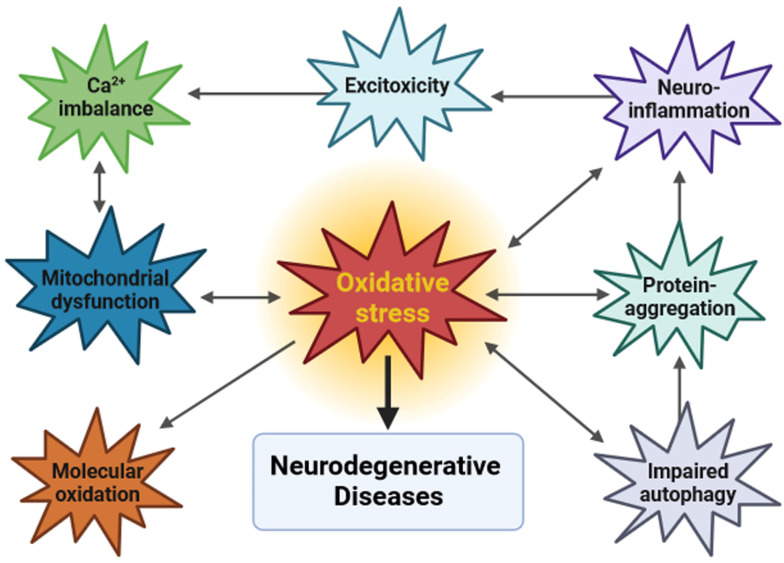
Schematic representation of the role of oxidative stress and other factors in the etiology and pathogenesis of NDs.

Several closely interlinked factors must be taken into account to understand the etiology and pathogenesis of NDs (Figure 1).

One of the major dysfunctions that characterizes most if not all NDs is an increased oxidative stress that occurs when there is an imbalance between the production of reactive oxygen/nitrogen species (ROS/RNS) and the neuronal antioxidant defenses, either enzymatic, such as superoxide dismutase (SOD) and catalase, or non-enzymatic, such as glutathione [3]. ROS play a physiological role in cell signaling and in the defense of cells against microorganisms, but their excessive production can cause serious damage due to the uncontrolled oxidation of different biomolecules such as proteins, DNA, and lipids, leading to cellular stress that can evolve to cell death [4]. 

Neurons are very sensitive to oxidative damages for several reasons. Firstly, they have high oxidative phosphorylation because they must produce high amounts of adenosine triphosphate (ATP) to maintain the homeostasis of ions involved in neurosecretion and neurotransmission [5,6]. Neurons therefore undergo high O_2_ consumption, thereby having a greater propensity to form ROS with respect to other cell types. A second important aspect is that many transition metal ions (in particular, iron) accumulate into neurons, which can further catalyze radical oxygen species through Fenton’s reaction [7]. Thirdly, neuronal membranes are rich in polyunsaturated fatty acids that can be easily oxidized and thus alter their permeability and membrane plasticity [8]. Finally, neurons contain fewer antioxidant defenses than other cells [4]. Taken together, all these factors, which tend to worsen with aging, contribute to the neuronal overproduction of ROS driving the occurrence of NDs.

Since the main source of ROS is the mitochondrion, it is reasonable to assume that the first cellular damage occurs at the mitochondrial level and leads to mitochondrial dysfunctions [9]. Whether ROS are the cause or the effect of mitochondrial dysfunction is difficult to determine, but the two mechanisms are certainly interdependent in the pathogenesis of NDs. Mitochondria are highly dynamic organelles that maintain their integrity and quantity through fusion and fission events which, in addition to producing ATP, regulate Ca^2+^ homeostasis and cell death. Several studies have shown that in NDs there is a widespread mitochondrial fragmentation, an altered distribution of the organelles in cell bodies and neuronal processes, and altered contacts between mitochondria and the endoplasmic reticulum (ER) [10,11]. Mitochondrial dysfunction is tightly related to aberrant Ca^2+^ signaling [12]. Since 1984, it has been hypothesized that a derangement of intracellular Ca^2+^ signaling is linked to neurodegeneration, and subsequent studies, both in cellular and animal models, have confirmed that in all NDs there is an altered Ca^2+^ homeostasis in neurons [13,14,15]. 

In addition, oxidative stress also plays a role in the activation of the autophagy process [16] which, in turn, contributes to the removal of ROS, damaged mitochondria, and aggregated proteins from cells. In NDs, the autophagy mechanism is impaired, leading to the accumulation of misfolded or aggregated proteins, and the formation of amyloid fibrils, characteristic of AD, PD, and other NDs [17].

Following acute or chronic neuronal injury, microglia release pro-inflammatory cytokines such as Tumor Necrosis Factor-α (TNF-α), Interferon gamma-γ, ROS, nitric oxide (NO), increasing neuroinflammation, redox imbalance, and aggregated proteins in the brain [18]. In addition, glial cells play a key role in maintaining the excitation–inhibition (E/I) balance necessary for proper neuronal function and the maintenance of synaptic plasticity within the CNS. In degenerative processes, the E/I imbalance leads to alterations in the glutamatergic and GABAergic neurotransmitter systems, resulting in hyperexcitability and altered Ca^2+^ levels [19].

We can thus conclude that oxidative stress and inflammation feed off each other, constituting an important pathological loop which is a central point to the progression of neurodegeneration.

## 2. Current Therapeutic Approaches to NDs

Despite the fact that NDs may occur as inherited familial forms associated with genetic mutations, most of them have a sporadic onset. This underlines the importance of environmental factors, regardless of genetic predisposition. Due to their complex and heterogeneous causes, NDs remain incurable despite the many pharmacological therapies available on the market. Currently approved drugs are mainly aimed at relieving symptoms and do not address the primary causes [20]. Another problem in finding effective drugs targeting NDs is the ability of active molecules to efficiently cross the blood–brain barrier [21]. The most widely used therapies in NDs are based on the manipulation of enzyme activities and neurotransmitter synthesis. 

AD is one of the most devastating NDs, affecting millions of people worldwide [22]. The pathological features of AD are the presence of amyloid plaques and aggregates of the neurofilament protein tau in the brain, leading to neuronal death and loss of synapses, resulting in memory loss. Many drug discovery studies in AD have been based on the ‘amyloid hypothesis’ as the main cause of the disease, and led to the development of drugs that counteract the formation of Aβ peptides, e.g., α,β secretase inhibitors, which reduce the cleavage of β-amyloid precursor protein (APP) and decrease Aβ40 or Aβ42 peptide generation, both considered to be neurotoxic and thus responsible of neuronal loss [23]. Other therapies focus on the treatment of hyperphosphorylated tau protein aggregates, that are a histopathological hallmark of AD and other related tauopathies [24], or on more generic targets such as neuroinflammation and neuronal mitochondrial dysfunction [25].

PD is the second most common neurodegenerative disease in the world. It is characterized by the presence of α-synuclein aggregates in inclusions called Lewy bodies, which cause the degeneration and death of dopaminergic neurons in the substantia nigra. The resulting loss of neurotransmitters is involved in the development of bradykinesia, resting tremor, muscle rigidity and postural instability [26]. Therapeutic treatments have therefore focused on dopamine replacement to alleviate the symptoms of the disease; these include receptor agonists such as apomorphine, dopamine agonists such as levodopa, and monoamine oxidase inhibitors [27]. Nevertheless, long-term chronic treatment with receptor agonists leads to dopaminergic sensitization with a worsening of the disease [28].

ALS is a progressive and fatal neurodegenerative disease characterized by the injury and selective death of motor neurons in the spinal cord, brainstem, and cerebral cortex. The etiology of ALS is highly multifactorial and is associated with glutamate-mediated excitotoxicity, oxidative stress, inflammation, loss of neurotrophic factors, protein misfolding and aggregation, and mitochondrial dysfunction [29]. Due to the complexity and heterogeneity of the disease spectrum, ALS remains incurable. Currently approved drugs, such as the anti-glutamatergic Riluzole, the antioxidant molecules Selegiline, Rasagiline, and vitamin E, and the newer Edaravone, are able to moderately slow down motor neuron degeneration by reducing oxidative stress and by enhancing free radical neutralization. Clinical trials are ongoing to validate new drugs with innovative mechanisms of action and new pharmacological targets. These new drugs have been clearly reviewed by Tzeplaeff and coauthors [30]. 

Many current therapies for the treatment of various neurodegenerative diseases have proven to be ineffective in a high percentage of patients [23]. This can be explained by the fact that NDs are complex neuropathologies caused by multiple pathogenetic mechanisms, some of which are related to the ageing of the human population while others are due to the increase in environmental risk factors. All this proves beyond any reasonable doubt that there is an urgent need to find other therapeutic molecules able to prevent or treat these diseases possibly before the onset of symptoms.

## 3. Essential Oils and Neurodegenerative Diseases

Essential oils (EOs) are complex mixtures that can consist of as many as 100 different molecules, consisting of aromatic and aliphatic compounds. The name “essential” was given because they were believed to capture a plant’s essence, that is, its odor and flavor. EOs are extracted via steam distillation or other methods (e.g., cold extraction) from plants in which they act mainly as insecticidal, parasiticidal, antimicrobial, and antioxidant agents. 

In animals, EOs play a role as antioxidant, anti-inflammatory, analgesic, antinociceptive, and anticancer mixtures; therefore, EOs have been used in traditional medicine, and in the pharmaceutical, cosmetic, and food industries [31].

Several studies have been performed to evaluate the EOs’ therapeutic activities; however, the high variability of their formulations often makes it difficult to understand which of the several active molecules are responsible for their effects. For these reasons, studies are increasingly oriented towards essential oils characterized by a high titer of a single compound, or directly towards individual purified compounds, which allows a more traditional pharmacological approach and more reproducible results [32,33].

EO molecules can be classified in different groups: terpenoids, phenylpropenes (such as eugenol, cinnamaldehyde, and safrole), terpenes (such as functionalized derivatives of alcohols (geraniol, α-bisabolol), ketones (menthone, p-vetivone) aldehydes (citronellal, sinensal), esters (γ-tepinyl acetate, cedryl acetate), and phenols (thymol)). Interestingly, almost all these molecules have shown to be multitarget, acting on different cellular pathways and molecular targets at the same time. 

The antioxidant activity of EO molecules is quite complex and is due to the inherent ability of some of their components, particularly phenols, to stop or delay the aerobic oxidation of organic matter, although the procedure by which the oil is obtained (essentially, distillation) limits the content of phenolics in the final matrix. Phenol-free EOs also display antioxidant behavior. This could be due to the presence of double bonds and to the radical chemistry of some terpenoids (e.g., eucalyptol) and other volatile constituents (e.g., sulfur-containing components) [33,34], and has been confirmed in vitro by using different biological and nonbiological methods, such as the 1,1-diphenyl-2-picrylhydrazyl (DPPH), 2,2-azinobis [3-ethylbenzthiazoline]-6-sulphonic acid (ABTS), and hydrogen peroxide (H2O2) radical scavenging capacity assays, and the inhibition of lipid peroxidation by measuring the thiobarbituric acid reactive substances (TBARs) [35,36]. The antioxidant effect of sweet orange essential oil, whose main components are D-limonene (usually >85%) octanal and decanal (usually 1–2%), has been demonstrated on isolated brain homogenates via the inhibition of Fe^2+^-induced lipid peroxidation [37]. Many different in vitro studies have been carried out by using EOs or their molecules, such as cinnamaldehyde, eugenol, carvacrol, or β-caryophyllene, with interesting results. 

Moving to in vivo studies, some interesting results were obtained using the model organism *Caenorhabditis elegans*. In *C. elegans*, Yen and coauthors have demonstrated the antioxidant properties of EO from *Zelkova serrata*, very rich in 7-hydroxycalamenene (>81%) [38]. In these worms, both the juglone treatment and heat shock induced oxidative stress, increased cytosolic ROS, and decreased survival. *Z. serrata* EO treatment reversed all these effects. Moreover, a similar protective effect was found in CL4176 nematodes treated with Aβ peptide, suggesting that this specific EO could be used as a drug in the treatment of AD. Rose essential oil extracted from *Rosa rugosa* cultivars contains high amounts of citronellol, geraniol, and octadiene. These EOs possess anti-Aβ, anti-oxidative, and anti-depression-like properties, demonstrating neuroprotective potential in *C. elegans*. Moreover, in *C. elegans* treated with 6-Hydroxydopamine hydrobromide (6-OHDA), a neurotoxin that damages the dopaminergic neurons and is thus considered a PD model, rose EOs reduced α-Syn aggregations and diminished dopamine neuron degenerations, reversing the food-sensing behavioral disabilities induced by the 6-OHDA treatment, and prolonging the lifespan of the nematode [39]. In this study, an increased SOD-3 activity in neurons was also observed, that has been linked to the anti-oxidative effect of rose EO that is indeed capable to reduce internal cellular ROS levels [39]. The same rose EO was tested in an AD model based on transgenic CL4176 *C. elegans* which overexpresses the human Aβ1−42 gene when the temperature rises from 15 to 25 °C. In these worms, rose EO significantly inhibited AD-like symptoms, such as worm paralysis and hypersensitivity to exogenous 5-hydroxytryptamine, in a dose-dependent manner. Indeed, rose EO significantly suppressed Aβ deposits and reduced the Aβ oligomers, alleviating the toxicity induced by Aβ overexpression. Interestingly, the authors verified that the major components of rose EO alone (β-citronellol and geraniol) were less effective than the oil itself [40].

The essential oils of *Cinnamomum* species, whose main component is cinnamaldehyde, have demonstrated to have neuroprotective effects in a mouse model of PD [41], protecting neurons against dopaminergic cell death, decreasing striatal neurotransmitter dysregulation, and finally improving motor deficits in these mice. The mechanisms proposed for cinnamon EOs included autophagy regulation, antioxidant effects, the upregulation of Parkin, DJ-1, and glial cell-derived neurotrophic factor, as well as the modulation of the Toll-like Receptor/Nuclear Factor-kB pathway, with the consequent inhibition of the excessive brain proinflammatory responses. In addition, in vitro and in vivo studies have shown that cinnamon extracts may cross the blood–brain barrier and positively affect the oligomerization process and aggregation of α-synuclein [41]. A well-characterized cinnamon EO (cinnamaldehyde 81%; cinnamyl acetate 4%) was shown to act in vitro as a monoamine oxidase (MAO A and MAO-B) inhibitor [42], preventing the extensive hydrolysis of monoamine neurotransmitters (serotonin, dopamine, norepinephrine, etc.) and thus opening up the possibility to use this EO as a modulator of amine metabolism in PD and AD. 

Another in vitro study analyzed the effect of a characterized EO from *Aloysia citrodora* (containing D-limonene, geranial, neral, eucalyptol, curcumene, spathulenol, and caryophyllene oxide) in a AD model based on a neuroblastoma cell line treated with hydrogen peroxide or β-amyloid to induce neurotoxicity. This EO displayed effective antioxidant activities, radical-scavenging activities, and significant protective activities against both hydrogen peroxide- and β-amyloid-induced neurotoxicity with a mechanism involving iron-chelation [43].

An EO obtained by the plant *Ferulago angulata* containing as major components α-pinene (24%), β-pinene (23%), β-phellandrene (21%), and α-phellandrene (12%) was used to treat rats injected with scopolamine to simulate AD. This EO, vaporized into aromatherapy chambers, improved memory function with a mechanism involving the reduction in the oxidative stress in the rat hippocampus [44].

In a rat model of AD based on intracerebroventricular administration of amyloid-β peptide 1–42 (Aβ1–42), the inhalation of an EO of *Tetraclinis articulata*, containing α-pinene (23%), L-bornyl acetate (17%), camphor (15%), limonene (7%), β-myrcene (3%), and camphene (3%), was able to improve memory deficits by modulating acetylcholinesterase activity and by reducing oxidative stress in the rat hippocampus [45].

The oral administration of the EO of *Pulicaria undulata*, characterized by a high amount of carvotanacetone (>80%), followed by chrysanthenone (6%) and linalool (4%), was able to reverse toxicity in rats pretreated with rotenone, which mimics Parkinson’s disease [46]. Rotenone increases nitric oxide (iNOS activity), which in turn increases Interleukin-(IL)-1-β and TNF-α production, triggering an inflammatory response that leads to the degeneration of nigrostriatal dopaminergic neurons associated with accumulation of α-synuclein positive inclusions. Moreover, rotenone increases oxidative stress and finally cause dopaminergic neuronal loss in PD rats. *Pulicaria undulata* EO reduced both the expression of iNOS and of α-synuclein; thus, it could be considered as a potential anti-PD drug.

Two EOs from *Rosa damascena* and *Lavandula angustifolia*, with a high titer in lianalool, linalilatsetat, camphor, and eucalyptol, were administered via i.p. injections in mice treated with L-dopa and benserazide to induce toxic effect on dopaminergic neurons. The EOs acted as antioxidants, reducing oxidative stress biomarkers such as malondialdehyde, protein carbonyl content, and nitric oxide radicals in the plasma and brain of the treated mice [47]. This is an interesting result, since it has been demonstrated that in PD patients treated for a long time with L-dopa, the oxidative stress increases and promotes disease progression [48].

**Figure 2:**
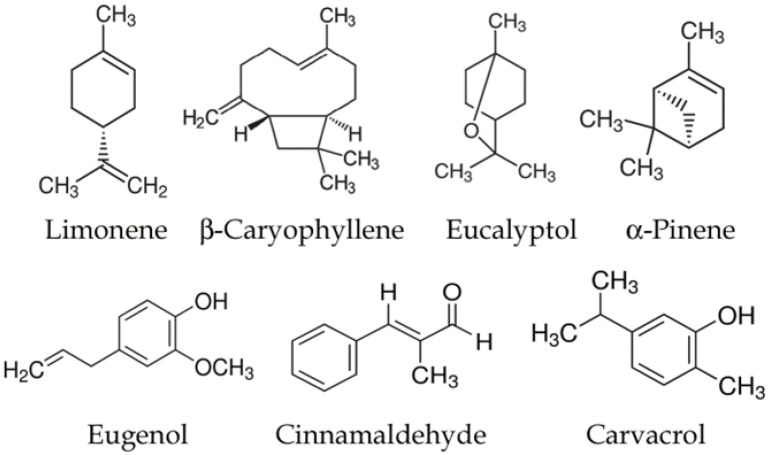
Structure of the main molecules with antioxidant effects in neurodegenerative models.

Single compounds isolated for essential oils can also have neuroprotective effects, as shown by several studies. The most studied EO molecules for NDs are shown in Figure 2.

Our research group recently found that eugenol administered orally or intravenously to rats is able to efficiently cross the blood–brain barrier. Moreover, in differentiated PC12 cells, an in vitro model that mimics PD because these cells synthesize and store catecholamines and dopamine, eugenol treatment increased cell survival and enhanced dopamine secretion [49]. Eugenol was also tested in a mouse model of PD based on the i.p. administration of 1-methyl-4-phenyl-1,2,3,6-tetrahydropyridine (MPTP). Remarkably, oral pre-treatment with eugenol was able to reduce the motor dysfunction caused by MPTP, while post-treatment with eugenol at a high dose worsened these symptoms. Attenuated levels of lipid peroxidation were found with eugenol pre-treatment while lipid peroxidation increased with eugenol post-treatment. This study underlines the difference between protective and therapeutic approaches, and shows that eugenol has a strong protective effect, but does not seem to have curative ones, at least not in this PD model [50]. In a previous study, the effect of orally administered eugenol was observed in a PD mouse model based on 6-hydroxydopamine (6-OHDA) intracerebroventricularly injected [51]. This study concluded that eugenol was effective in ameliorating the behavioral impairments in PD mice, and it was able to increase antioxidant activities in the striatum. Eugenol was also tested in a 5× familiar AD (5×FAD) mouse model. The results of this study indicate that eugenol oral treatment effectively mitigated cognitive impairments in 5×FAD mice, decreasing neuronal cell loss and Aβ deposition [52]. The underlying mechanism of action seems to be related to the inhibition of necroptosis and to the increase in microglial phagocytosis, which were responsible for the observed reduction in neuronal cell loss and Aβ deposition, respectively. 

Cinnamaldehyde, the major component of cinnamon EO, was tested (i.p. administered) in an AD model based on intracerebroventricular streptozotocin injection. The results showed that this compound improved recognition/spatial memory deficits and anxiety-like behavior in the treated rats. In addition, cinnamaldehyde affected Aβ aggregation and caspase-3 cleavage in the hippocampus, suggesting its involvement in the regulation of hippocampal Insulin Receptor Substrate (IRS)-associated phosphoinositide 3-kinase (PI3K)/serine/threonine-protein kinase (Akt)/glycogen synthase kinase-3β (GSK-3β) and caspase-3 pathways in this sporadic AD rat model [53]. In 5×FAD mice, cinnamaldehyde treatment (i.p. administered) led to an improvement in AD symptoms by reducing β-site APP Cleaving Enzyme 1 (BACE1) levels through the activation of the Silent information regulator 1 (SIRT1)-peroxisome proliferator-activated receptor γ (PPARγ) coactivator 1α (PGC1α)-PPARγ pathway, suggesting that it might be a useful therapeutic approach for AD patients [54]. Finally, in an aluminum-induced AD rat model, cinnamaldehyde (orally administered) reduced the loss of dendritic spines, neurofibrillary degeneration, and the appearance of neuritic plaques, with a concomitant improvement in memory and the intellectual performance of the treated animals [55]. Cinnamaldehyde was also tested in an MPTP mouse model of PD, characterized by selective dopaminergic neuronal death in the substantia nigra. In this model, cinnamaldehyde (i.p. administered) prevented neurodegeneration by modulating the autophagy process [56].

D-limonene, in primary rat cultures treated with the neurotoxic peptide Aβ1–42, showed protective effects by increasing neuronal viability and reducing the amount of ROS [57]. In a Drosophila AD model, D-limonene treatment did not affect Aβ42 accumulation and aggregation; nevertheless, it was able to prevent cell death and decrease reactive oxygen species levels, extracellular signal-regulated kinase phosphorylation, and inflammation in the brain or in the eye imaginal discs of Aβ42-expressing flies [58]. In a rotenone-induced PD model in rats, the oral administration of D-limonene significantly reduced dopaminergic neuronal loss in the substantia nigra, reduced oxidative stress, and normalized the expression of inflammatory mediators by modulating the altered Nuclear factor kappaB/Mitogen-Activated Protein Kinase (NF-κB/MAPK) signaling pathway in the brain of rotenone-treated rats [59]. Nevertheless, these suggestive results are difficult to understand in the light of D-limonene pharmacokinetics that does not support its ability to cross the blood–brain barrier [49].

β-caryophyllene, a terpene extracted from clove, hemp, and black pepper, has shown neuroprotective effects in rotenone-induced PD in rats. This effect has been linked to a reduced oxidative stress and lipid peroxidation in dopaminergic neurons [60]. β- caryophyllene has also been studied by using in vitro AD models. In PC-12 cells overexpressing amyloid-β protein precursor, β-caryophyllene exhibited the potential to dramatically increase PC-12 cell viability, inhibiting the Janus kinase 2-signal transducer and activator of transcription 3 (JAK2-STAT3)-BACE1 signaling pathway [61]. β-caryophyllene is also capable to positively modulate microglial inflammation, as assessed in vitro on primary microglia cells inflamed by LPS [62]. It has also been shown that the anti-inflammatory and immunomodulatory effects of this compound depend on its selective binding to the type two cannabinoid receptor [63]; interestingly, as a cannabinoid ligand, it is very likely that β-caryophyllene could cross the blood–brain barrier. 

Carvacrol is a phenolic monoterpene present in oregano and thyme EOs. Carvacrol in vitro was found to protect neuronal PC12 cells from toxicity induced by 6-hydroxydopamine (6-OHDA) administration, in a dose-dependent manner. Its mechanism of action at the cellular level appears to be linked to an increased cell viability, probably due to a reduction in intracellular reactive oxygen species and in intracellular lipid peroxidation. [64]. These antioxidant activities have been confirmed in SH-SY5Y neuronal cells, treated with hydrogen peroxide. In these experiments, the neuroprotective effects of the compound were evaluated by analyzing the expression of caspase-3 and the cholinesterase enzymatic activities. Carvacrol reduced caspase-3 expression and showed inhibitory activities against acetylcholinesterase and butyrylcholinesterase. These anti-enzymatic properties, associated with its antioxidant activity, underline its possible use as a coadjutant in preventing and treating AD [65]. In Wistar rats, orally administered carvacrol was able to counteract the effects of the intracerebroventricular (ICV) injection of amyloid Aβ1–42. Aβ-treated rats exhibited impaired long-term potentiation (LTP) induction in the dentate gyrus, but carvacrol ameliorated Aβ-associated changes in synaptic plasticity [66]. Carvacrol’s potential in managing and treating AD and PD has been recently clearly reviewed by Javed and coauthors [67]. 

Citronellol is a monoterpene found in the essential oils of plants of the Cymbopogon genus. It has been reported to possess hypotensive, analgesic, anti-diabetic, vasorelaxant, and anti-inflammatory properties [68]. Citronellol’s neuroprotective activities have been recently reported [69] against PD in a rat model based on rotenone administration. The experimental PD model was obtained by intraperitoneal injection of rotenone (2.5 mg/kg) once a day for four weeks, while citronellol was administered orally, dissolved in olive oil. Citronellol, if administered prior to rotenone exposure, reversed the free radical production measured in the midbrain of experimental animals, probably through its anti-oxidative properties. Nonetheless, citronellol diminished IL-6, IL-1β, TNF-α, and metalloproteinase 9 secretion in the midbrain. Also, iNOS activity was found to be reduced by citronellol. The activation of microglia and astrocytes increased in the striatum of the rotenone-treated animals, and citronellol administration significantly diminished this activation. Finally, citronellol significantly reduced dopaminergic neuronal loss and prevented the over-expression of α-synuclein in the rotenone-intoxicated rats. At a molecular level, the activity of citronellol is certainly multitarget, and two of them involve apoptosis modulation and mTOR pathway modulation in dopaminergic neurons. 

In vitro studies testing EOs or EO molecules on cellular ND models are summarized in Table 1 while animal models of NDs are summarized in Table 2.

## 4. Conclusions

NDs are pathologies that are still lacking effective therapies. Today, there is a lack of drugs able to effectively slow down their progression after diagnosis or to prevent their onset in subjects with an increased genetic or environmental risk. EOs are an almost inexhaustible source of molecules with different activities at a cellular level, which include antioxidant and anti-inflammatory effects. These proprieties could be effectively exploited for slowing down or blocking neurodegenerative processes. Thus, EOs could be used to develop nutraceutical formulations, rather than drugs, for the treatment of NDs. This makes it necessary to evaluate their safety profiles for their chronic administration, especially in frail and elderly patients. EOs are complex mixtures which can number over 100 different molecular components. Many studies demonstrate that there can be synergistic actions between different molecules present in a single EO. On the other hand, the lack of homogeneity in their composition makes it very difficult to standardize their therapeutic use. Moreover, while the safety profile of individual EO molecules is easier to evaluate and for many of these compounds it has been already established, safety data are still lacking for many EOs. For all these reasons, we have proposed the use of single EO molecules, which can be individually characterized from the point of view of activity, pharmacokinetics, and toxicity, and eventually mixed afterwards to obtain combined or synergistic activities. Interestingly, some EO molecules can easily cross the blood–brain barrier, exerting their strong anti-inflammatory and antioxidant activities in the brain with an overall low toxicity. EO molecules have almost always been shown to possess multitarget actions at the cellular and molecular level. Even if most studies on EOs have been performed on post-symptomatic models, we believe that their characteristics make these compounds particularly appropriate for the prevention of NDs. EO molecules at safe doses could be chronically administered to the elderly population or to individuals carrying ND-related mutations as substances able to prevent or delay the development of NDs. However, controlled clinical trials are needed to confirm this possibility and the promising results obtained by these molecules both in vitro and in preclinical rodent models.

## Figures and Tables

**Table 1:** In vitro studies on the effect of essential oils in neurodegeneration models.

Essential Oil/Eo Single Compound	Disease Model	Effects	References
cinnamaldehyde	PD: 6-OHDA induced apoptosis in PC12 cells	Decreased cyt-c Increased surviving Reduced p-p44/42/p44/42 levels Reduced cytotoxicity	[41]
cinnamaldehyde	PD: MPP+ induced neurotoxicity in human neuroblastoma BE(2)-M17 cell	Recovery of MPP+ induced cell death	[41]
*Cinnamomum zeylanicum*	PD and AD models: enzymatic assays	Monoamine oxidase (MAO A and MAO-B) inhibition	[42]
*Aloysia citrodora*	AD model: neuroblastoma cell line treated with hydrogen peroxide or β-amyloid	Antioxidant Radical-scavenging activity Protection against β-amyloid-induced neurotoxicity	[43]
eugenol	Neuronal cell model	Increased cell survival Enhanced dopamine secretion	[49]
D-limonene	AD model: primary rat cultures treated with the neurotoxic peptide Aβ_1–42_	Increased neuronal viability Antioxidant	[57]
β-caryophyllene	AD model: PC-12 cells overexpressing amyloid-β protein precursor	Increased cell viability	[61]
β-Caryophyllene	LPS-Induced Primary Microglia M1/M2 Imbalance	Anti-inflammatory	[62]
carvacrol	PD: PC12 cells treated with 6-hydroxydopamine (6-OHDA)	Increased cell viability	[64]
carvacrol	AD: SH-SY5Y neuronal cells treated with hydrogen peroxide	Inhibition of acetylcholinesterase and butyrylcholinesterase	[65]

**Table 2:** In vivo studies on the effect of essential oils in neurodegeneration models.

Essential Oil/EO Single Molecule	Disease Model	Effects	References
*Zelkova serrata*	Oxidative stress and heat shock induced in *C. elegans*	Increased stress resistance Decrease in ROS	[38]
*Zelkova serrata* (1S,4S-7-hydroxycalamenene)	AD model: Aβ induction *in C. elegans* (CL4716 strain)	Decrease in Aβ-induced toxicity Decrease in ROS	[38]
*Rosa setate × Rosa rugosa*	PD model: *C. elegans* (BZ555 strain for dopaminergic neurotoxicity, OW13 strain for α-synuclein expression)	Reduction in α-synuclein accumulation Decrease in dopaminergic neuron degeneration Decrease in ROS	[39]
*Rosa setate × Rosa rugosa*	*C. elegans* CF1553 strain (expressing antioxidant enzymes)	Increase in SOD-3 expression Decrease in ROS	[39]
*Rosa setate × Rosa rugosa*	AD model: *C. elegans* CL4176, CL2355 (Aβ inducible) CL2006 (Aβ constitutively expressed)	Delay of AD-like symptoms Suppression of Aβ	[40]
cinnamaldehyde	PD model: mice treated with 1-metil 4-fenil 1,2,3,6-tetraidro-piridina	Autophagy regulation, antioxidant effects, upregulation of Parkin, DJ-1 upregulation of glial cell-derived neurotrophic factor, modulation of the TLR/NF-κB	[41]
*Ferulago angulata*	AD model: rats treated with scopolamine	Improved memory function	[44]
*Tetraclinis articulata*	AD model: intracerebroventricular administration amyloid-β peptide 1–42	Improved memory function	[45]
*Pulicaria undulata*	PD model: rat treated with rotenone	Decrease in iNOS activity Decrease in α-synuclein	[46]
*Rosa damascena*	PD model: mice treated with L-dopa and benserazide	Reduction in oxidative stress biomarkers (malondialdehyde, protein carbonyl content, and nitric oxide radicals	[47]
*Lavandula angustifolia*	PD model: mice treated with L-dopa and benserazide	Reduction in oxidative stress biomarkers (malondialdehyde, protein carbonyl content, and nitric oxide radicals	[47]
eugenol	PD model: mice treated with 1-methyl-4-phenyl-1,2,3,6-tetrahydropyridine (MPTP)	Reduced motor disfunction (pretreatment) Worsening of motor disfunction (post-induction treatment)	[50]
eugenol	PD model: intracerebroventricularly injected 6-hydroxydopamine	Improvement in behavioral impairments Antioxidant activity in the striatum	[51]
eugenol	AD model: 5 × FAD mice	Decreased neuronal cell loss Decreased Aβ deposition	[52]
cinnamaldehyde	AD model: intracerebroventricular streptozotocin injection in rats	Improved recognition/spatial memory Inhibition of Aβ aggregation	[53]
cinnamaldehyde	AD model: 5 × FAD mice	Improvement of AD symptoms	[54]
cinnamaldehyde	AD model: rat treated with aluminum	Reduced loss of dendritic spines Reduced neurofibrillary degeneration Improvement in memory and intellectual performance	[55]
cinnamaldehyde	PD model: mice treated with MPTP	Prevention of neurodegeneration	[56]
D-limonene	AD model: Aβ42-expressing drosophila	Prevention of cell death Extracellular signal-regulated kinase phosphorylation Decrease in ROS Decrease in inflammation	[58]
D-limonene	PD model: rats treated with rotenone	Reduced dopaminergic neuronal loss Reduced inflammatory markers	[59]
β-caryophyllene	PD model: rats treated with rotenone	Prevention of the loss of dopaminergic neurons Reduction in lipid peroxidation Increased activity of antioxidant enzymes Decrease in inflammatory markers Decrease in activated astrocytes and microglia	[60]
Carvacrol	AD model: intracerebroventricular injection of amyloid Aβ1–42 in rats	Improved Aβ-associated impairments in synaptic plasticity	[66]
Citronellol	PD model: rats treated with rotenone	Reduced dopaminergic neuronal loss Prevented the over-expression of α-synuclein	[69]
